# Low-Cost, High-Yield Zinc Oxide-Based Nanostars for Alkaline Overall Water Splitting

**DOI:** 10.1021/acsomega.3c03958

**Published:** 2023-09-29

**Authors:** Gisella Maria Di Mari, Maria Chiara Spadaro, Francesco Salutari, Jordi Arbiol, Luca Bruno, Giacometta Mineo, Elena Bruno, Vincenzina Strano, Salvo Mirabella

**Affiliations:** † Dipartimento di Fisica e Astronomia “Ettore Majorana”, Università degli Studi di Catania, via S. Sofia 64, 95123Catania, Italy; ‡ CNR-IMM, Catania (University) Unit, via S. Sofia 64, 95123 Catania, Italy; § Dipartimento SIMAU, Università Politecnica delle Marche, Piazza Roma 22, 60121 Ancona, Italy; ∥ Catalan Institute of Nanoscience and Nanotechnology (ICN2), CSIC and BIST, Campus UAB, 08193 Bellaterra (Barcelona), Catalonia, Spain; ⊥ ICREA, Pg. Lluís Companys 23, 08010Barcelona, Catalonia, Spain

## Abstract

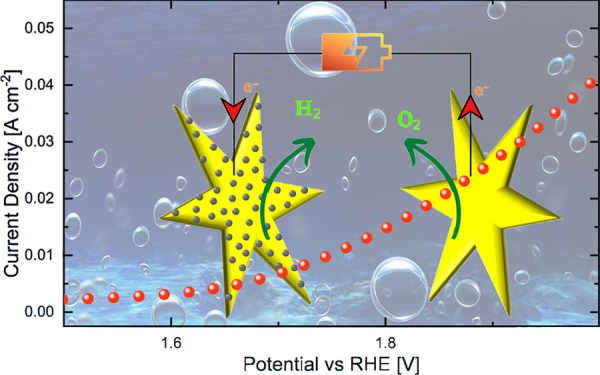

The investigation of high-efficiency and sustainable electrocatalysts for hydrogen evolution reaction (HER) and oxygen evolution reaction (OER) in alkaline media is critical for renewable energy technologies. Here, we report a low-cost and high-yield method to obtain ZnOHF-ZnO-based 2D nanostars (NSs) by means of chemical bath deposition (CBD). The obtained NSs, cast onto graphene paper substrates, were used as active materials for the development of a full water splitting cell. For the HER, NSs were decorated with an ultralow amount of Pt nanoparticles (11.2 μg cm^–2^), demonstrating an overpotential of 181 mV at a current density of 10 mA cm^–2^. The intrinsic activity of Pt was optimized, thanks to the ZnO supporting nanostructures, as outlined by the mass activity of Pt (0.9 mA mg_Pt_^–1^) and its turnover frequency (0.27 s^–1^ for a Pt loading of 11.2 μg cm^–2^). For the OER, bare NSs showed a remarkable result of 355 mV at 10 mA cm^–2^ in alkaline media. Pt-decorated and bare NSs were used as the cathode and anode, respectively, for alkaline electrochemical water splitting, assessing a stable overpotential of 1.7 V at a current density of 10 mA cm^–2^. The reported data pave the way toward large-scale production of low-cost electrocatalysts for green hydrogen production.

## Introduction

The transition toward net-zero emission within 2050 requires an unprecedented transformation of the energy production strategy.^1^ Hydrogen will certainly have a leading role in this transformation, as it has a huge energy content in terms of energy density, 120 MJ kg^–1^ (33.3 kW h kg^–1^), well higher than that of gasoline (44.4 MJ k kg^–1^).^1^ The chance to use hydrogen as a next future fuel is connected to the possibility of producing it in huge quantities by means of sustainable methods.^2,3^ One of the most efficient and sustainable approaches is electrochemical water splitting, by which water is converted into hydrogen and oxygen molecules through the hydrogen evolution reaction (HER) which occurs at the cathode and the oxygen evolution reaction (OER), which occurs at the anode.^4,5^ The HER process, in acid or alkaline media ([ ]), occurs through a reaction which involves 2 electrons:^6^
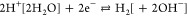
1

Traditionally, HER mechanism is assumed to proceed by the initial formation (Volmer step) of a hydrogen intermediate (denoted as H_ads_) which is formed via protons or water charge induced discharge, depending on the pH of the step:^6^
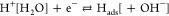
2

The Volmer step is then followed by a chemical recombination, named the Tafel step:^6^
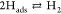
3

or by the transfer of a second electron in the Heyrovsky^6^
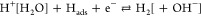
4

At the state-of-the-art, noble electrocatalysts such as Pt-group metals and Ru/Ir-based compounds are used as efficient electrocatalysts for the HER and OER, respectively, with very low overpotentials. Nonetheless, these materials are highly expensive and scarce,^7^ also classified as Critical Raw Materials (CRMs) since 2011 by the European Union.^8,9^ A solution to overcome the extensive noble metal utilization for water splitting is to develop proper substrates where ultralow amounts of the noble metal are efficiently utilized. In turn, this leads to a significant increase of the noble metal intrinsic activity.^10−12^

Acidic water electrolysis still lacks in the development of highly active and reasonably stable OER catalysts made of earth-abundant and low-cost materials.^13^ Hence, it is convenient to perform the electrochemical water splitting in an alkaline medium, where the range of effective HER and OER electrocatalysts is enlarged.^14−19^

Metal oxides composed of low-cost and earth-abundant elements are promising candidates as catalysts for water splitting purposes. Compared with other types of metal compounds, one of the advantages of metal oxides lies in the structural and compositional heterogeneity, which offers an electronic and crystal structure flexibility with various desirable physical/chemical properties.^20,21^ Among the others, zinc oxide (ZnO), an inorganic semiconductor largely abundant in nature and with good electrochemical activity, can be easily nanostructured in a multitude of morphologies by employing different methods.^22^ In particular, solution routes for ZnO nanostructure synthesis have a plethora of advantages, such as cost containment, simple laboratory setup, low-temperature processes, and fast kinetics growth.^23−27^ Nonetheless, ZnO has poor charge transfer aptitude and a high electron–hole recombination rate, which limits its direct application in electrocatalysis. A successful strategy to overcome such limitation is coupling ZnO nanostructures with noble metals, narrow-band semiconductors, and other materials, creating heterostructures with increased electron and hole mobility.^28,29^

Sun et al. reported a Ag-MoS_2_–ZnO, prepared by a double-step hydrothermal method. They tested the electrochemical activity for HER in alkaline media (0.1 M KOH) finding an overpotential of about 530 mV at 10 mA cm^–2^ for the bare ZnO and about 300 mV at 10 mA cm^–2^ for Ag-ZnO and MoS_2_–ZnO composites.^30^ Saini et al. reported a ZnO-MXene nanocomposite, obtained by a simple hydrothermal synthesis, with a variable ZnO content for electrocatalysis applications. The glassy carbon electrode with the bare ZnO as active material presents an overpotential higher than 700 mV at 10 mA cm^–2^ in acidic media.^31^

Menesez et al. reported a cobalt–zinc oxide catalyst for the OER, obtained by annealing treatments of the, respectively, hydroxide carbonate precursors.^32^ However, bare ZnO did not show any activity in alkaline media, as reported by the authors. Thus, while undecorated ZnO is not promising for water splitting application, there is a growing attention on ZnO nanostructures as proper and efficient support for active materials in electrocatalysts.

Here, we report a ZnO-based nanostructure, called nanostar (NS), obtained in high yield through an easy and green chemical bath deposition (CBD), showing promising performances for water splitting. After decoration with an ultralow amount of Pt, an overpotential of 181 mV at 10 mA cm^–2^ for HER was recorded, using 11.2 μg cm^–2^ of Pt. Also, an OER overpotential of 355 mV at 10 mA cm^–2^ in alkaline media was recorded for the bare NSs. Therefore, an alkaline full cell electrolyzer was set up fully based on ZnO NS (bare and Pt-decorated as the anode and cathode, respectively), showing a current density of 10 mA cm^–2^ with a potential of 1.7 V for 24h. These data open the way to ZnO-based full electrolyzers for water splitting.

## Materials and Methods

### Synthesis of ZnOHF-ZnO NSs and of Pt Nanoparticles

ZnOHF-ZnO NS powders were synthesized through CBD using a bain-marie configuration. The starting aqueous solution of 25 mM zinc nitrate hexahydrate, 25 mM hexamethylenetetramine, and 16 mM ammonium fluoride was kept at 90 °C for 10 min to obtain the NSs.^24,33^ The obtained nanostructures were washed 4 times in Milli-Q water and dried in an oven at 100 °C for 16 h. The powders were then dispersed in an aqueous solution of Milli-Q water.

In order to obtain a dispersion of Pt nanoparticles (NPs), we employed a green chemical reduction method under ambient conditions.^34^ Thirty microliters of 33 mM ascorbic acid aqueous solution, used as reducing agent, were added in 30 mL of 0.2 mM H_2_PtCl_6_ (Sigma-Aldrich, St. Louis, MO, USA, ≥ 99.9%) aqueous solution. The slightly yellow solution was then stirred at room temperature for 5 min and used without further treatments.

### Electrode Preparation

With the purpose of removing impurities from the substrates, graphene paper electrodes (GP, 1 × 1.8 cm^2^, 240 μm thick, Sigma-Aldrich, St. Louis, MO, USA) were washed with Milli-Q water and dried in N_2_. NSs were deposited through drop casting by using 35 μL of a 2 mg mL^–1^ NSs dispersion. Samples were then dried on a hot plate at 80 °C for 15 min. Pt NPs were deposited onto the NSs electrode through the NP dispersion drop addition onto NSs. Decorated samples are labeled as NSs-Pt.

**Figure 1:**
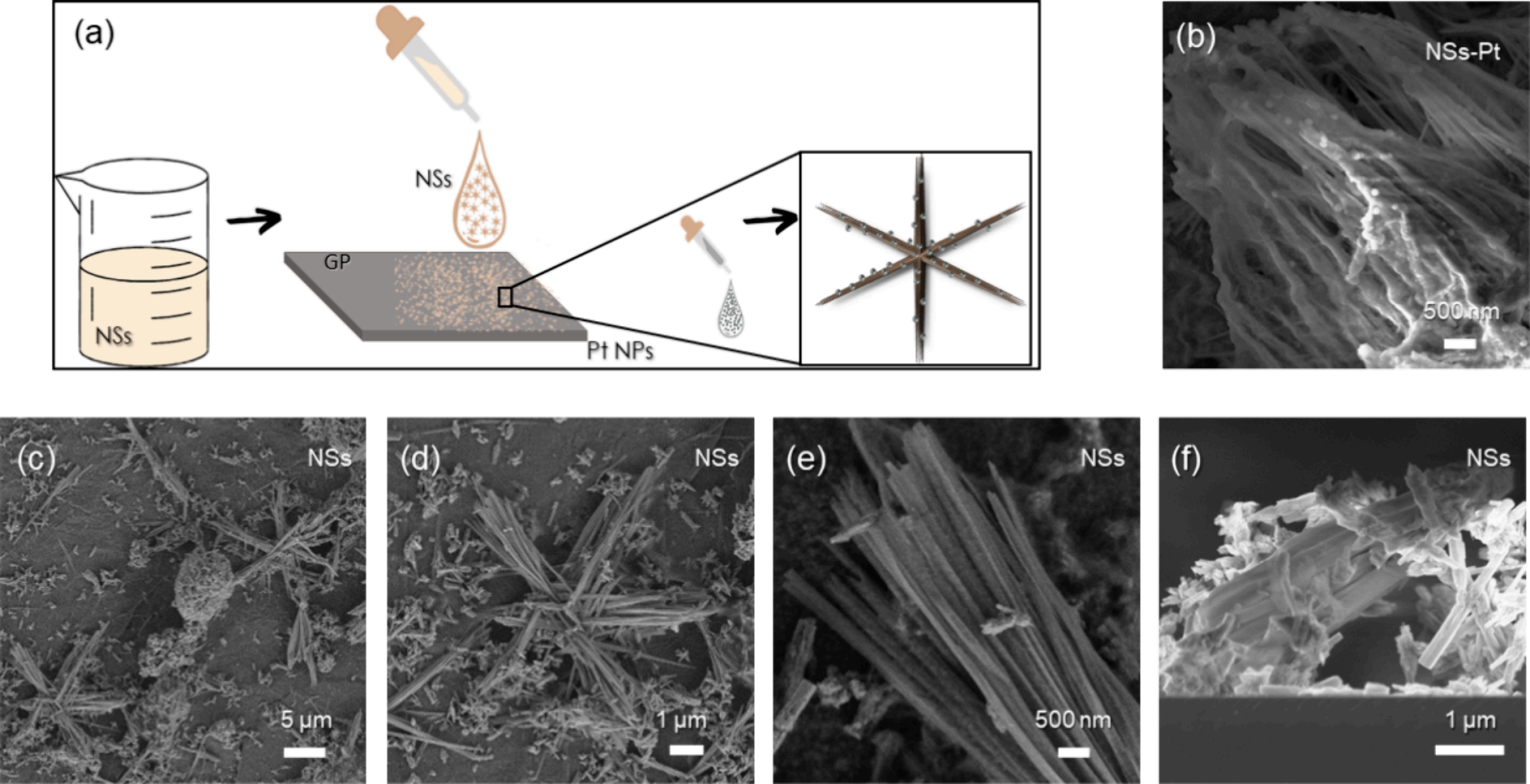
(a) Schematic of electrode preparation; (b) high-magnification SEM image of NSs-Pt; (c) NS SEM images at low, (d) medium, (e) and high magnification; and (f) NS cross section SEM image.

Figure 1a presents a schematic of the electrode preparation. We measured the mass of NSs on GP substrates through a Mettler Toledo MX5Microbalance and reported a value of (0.20 ± 0.01) mg.

### Pt-Decorated NS Characterization

Scanning electron microscopy (SEM, Gemini field emission SEM Carl Zeiss SUPRA 25, Carl Zeiss Microscopy GmbH, Jena, Germany) allowed us to analyze the surface morphology of bare and Pt-decorated nanostars. SEM images were further investigated through ImageJ software.^35^

High-resolution transmission electron microscopy (HRTEM) and scanning transmission electron microscopy (STEM) analyses were performed with a field emission gun FEI Tecnai F20 microscope. High-angle annular dark-field (HAADF) STEM was associated with electron energy loss spectroscopy (EELS) in a Tecnai microscope by using a GATAN QUANTUM energy filter to obtain compositional maps. STEM-EELS maps were performed using the O K-edge at 532 eV (blue), the Zn L edge at 1020 eV (green), Pt M edge at 2122 eV (red), and F K-edge at 685 eV (orange).

Rutherford backscattering spectrometry (RBS, 2.0 MeV He^+^ beam at normal incidence) allows us to evaluate the Pt amount on NSs. All RBS analyses were conducted with a 165° backscattering angle by using a 3.5 MV HVEE Singletron accelerator system (High Voltage Engineering Europa, the Netherlands). RBS spectra were then analyzed via the XRump software.^36^

All the electrochemical tests were carried out at room temperature by using a VersaSTAT4 potentiostat (Princeton applied research, USA) and a three-electrode setup with a Pt wire as the counter electrode, a KCl saturated calomel electrode (SCE) as the reference electrode, and the previously ZnO-based discussed electrodes as the working electrode (WE) using a 1 M KOH (ph 14, Sigma-Aldrich, St. Louis, MO, USA) solution as a supporting electrolyte. Cyclic voltammetry (CV) curves were acquired at a scan rate of 10 mV s^–1^ in the potential range −0.5 ÷ – 2 V vs SCE to stabilize the electrodes. Once stabilized, the catalyst HER activities were investigated by using linear sweep voltammetry (LSV) at a scan rate of 5 mV s^–1^ in the same potential region of CVs. We were able to perform electrochemical impedance spectroscopy (EIS) analyses by using a 10 mV sinusoidal voltage in the frequency range 104 ÷ 10–1 Hz, fixing the chosen potential for the measurement just after the onset potential (*E*_onset_, the minimum potential at which a reaction product is formed at the electrode). Mott–Schottky (M-S) analyses were performed on bare and decorated electrodes in the potential range of −1 ÷ – 0.15 V vs SCE, at 1000 Hz frequency.

### Pt Benchmark Sample

A Pt electrode was prepared as a benchmark for our electrode performances. A sputter apparatus Emitech K550X was used for the Pt depositions, in which the graphene paper substrate is located in the cathode, facing the Pt source (purity of 99.999%), at a distance of 40 mm and using Ar flux with a pressure of 0.03 mbar, and the substrate at room temperature. The deposition current was fixed at 50 mA, with a deposition time of 16 min. Both sides of the graphene paper were deposited.

### Electrochemical Data Analysis

The measured potentials (η′) were adjusted with the voltage drop compensation as follows:
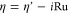
5

where *i* is the current and Ru [Ω] is the uncompensated resistance, extracted from the EIS data. We got current densities after the normalization to the sample geometrical area coated with the active material [1 × 1 cm], and the measured potentials vs SCE were converted to the reversible hydrogen electrode (RHE) using the well-known equation:^37^
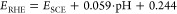
6

The turnover frequency (TOF) is described as the amount of product formed per unit time per active site:
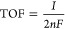
7

where *I* is the current measured at a fixed overpotential, 2 is the electron number involved in the HER process, *F* is the Faraday constant, and *n* is the number of active sites moles.^38^

Once the Pt amount loaded onto the electrodes is known through RBS analyses, the number of active Pt moles can be estimated as follows:
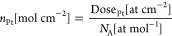
8

where *N*_A_ is Avogadro’s number and Dose_Pt_ is the RBS dose, describing the Pt atoms amount per cm^2^.

Lastly, the mass activity, which is a different way to report the intrinsic electrocatalyst activity, is described by the ratio between a certain current density value and the catalyst loading (obtained by multiplying *n*_Pt_ obtained through the RBS analysis for the Pt atomic weight):
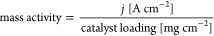
9

## Results and Discussion

NS morphology is shown in Figure 1c–e, reporting SEM images of NSs on GP substrates at low, medium, and high magnification, respectively. The star arms, each about 5 μm long and composed of batches of parallel wires, draw 6 angles of 60°, equally spaced on the plane. Figure 1f represents an NS cross section SEM image. From the morphological investigations, it is possible to appreciate the high surface to volume ratio of these peculiar nanostructures. This represents a huge advantage, especially if NSs are used as supports for electrocatalysts.

Figure S1 shows NSs onto the GP SEM image at low magnification.

After Pt decoration by drop casting, Pt NPs, 2 nm in size,^10^ were uniformly spread onto the NS sample. The Pt NPs tend to aggregate, forming bigger aggregates with dimensions of hundreds of nm, as shown in Figure 1b, reporting the SEM image of NSs-Pt at high magnification.

**Figure 2:**
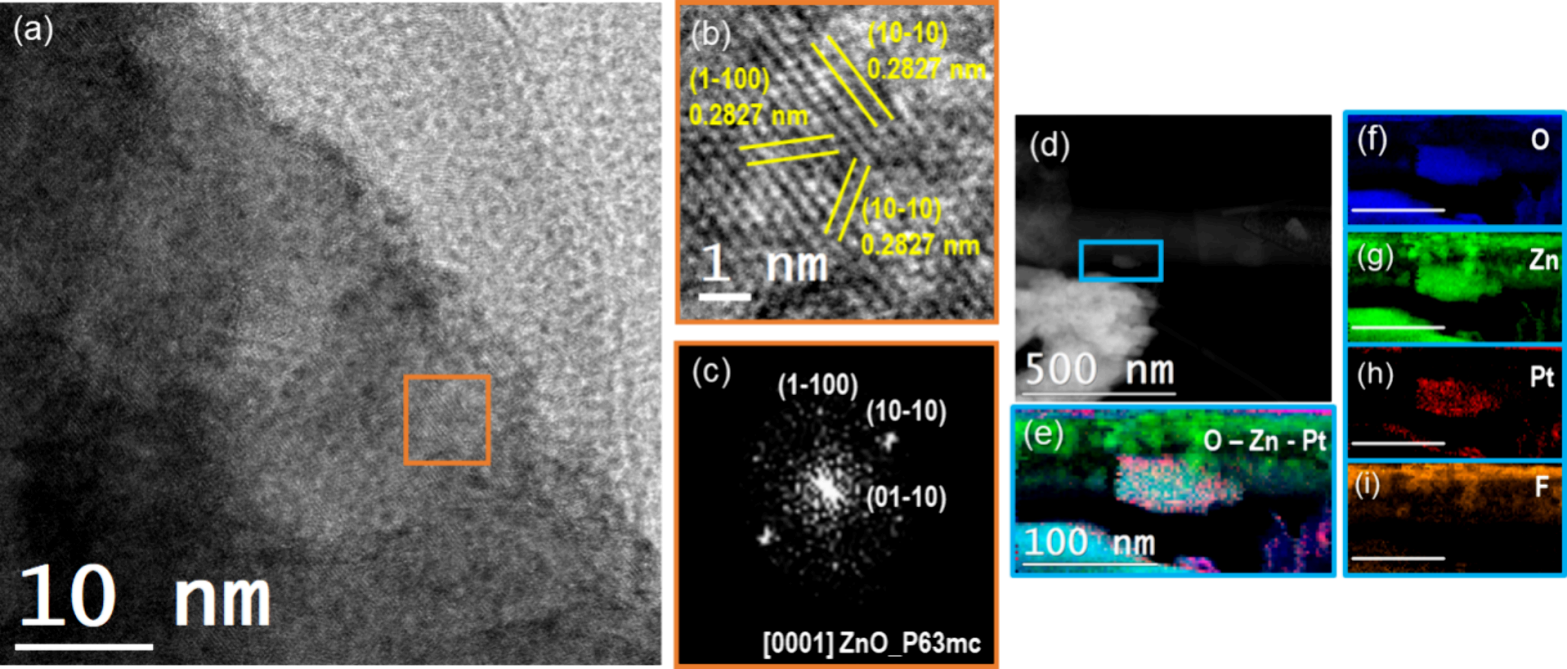
(a) HRTEM images showing the ZnO nanostar surface detail, together with (b) the orange box image magnification with the lattice spacings in yellow and (c) corresponding power spectrum (FFT) analysis. (d) STEM-HAADF micrograph showing the nanostar morphology and light blue box indicating the area where the EELS spectrum image map has been acquired. The resulting (e–i) STEM-EELS maps are shown evidencing the detected elements (scale bar 100 nm).

Transmission electron microscopy (TEM) has been exploited in order to gain deeper insight into the atomic structure of the NSs and the corresponding elemental distribution. High-resolution TEM (HRTEM) images evidenced that the NSs present a wurtzite hexagonal crystalline structure belonging to the P63mc (186) space group. In Figure 2a the NS is imaged along its [0001] axis parallel to the electron beam. Electron energy loss spectroscopy in scanning TEM mode (EELS-STEM) confirmed the chemical composition of the NSs, evidencing the presence of decorating NPs (Figure 2b). Residual fluorine is justified with the coexistence of the ZnOHF-ZnO mixed phase. ZnO-ZnOHF NSs X-ray diffraction analysis is reported in Figure S11, in which ZnO and ZnOHF PDF cards are reported below.

### Electrochemical Characterization

**Figure 3:**
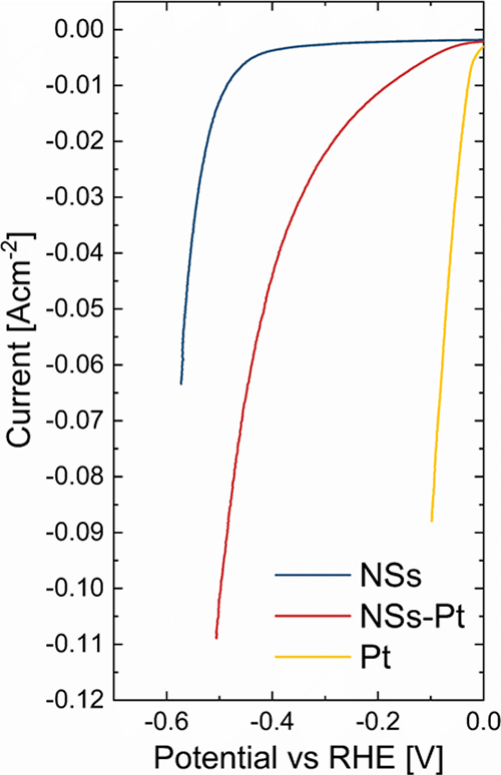
Polarization curves of bare NSs, NSs-Pt, and of Pt electrodes.

In order to perform a proper electrochemical evaluation of bare and Pt-decorated NSs in alkaline conditions, electrochemical analyses were performed in 1 M KOH (Figure 3, light blue and bordeaux lines respectively). The Pt sputtered electrode (yellow line) was used as Pt benchmark performance. All curves are corrected for the voltage drop, obtained from the EIS spectra, as reported in Figure S2. The polarization curves clearly showed that the presence of Pt NPs drastically lowers the activation energy barrier for the H_2_ production, and a strong variation in the overpotential at a constant current of 10 mA cm^–2^ was recorded as shown in Table 1.

**Table 1:** Overpotential Values of NSs, NSs-Pt, and Pt; TOF, Mass Activity, and Pt Loading Values of NSs-Pt

samples	overpotential (@10 mA cm^–2^) (mV)	TOF (@50 mV)	mass activity (@10 mA cm^–2^)	Pt loading
NSs	485			
NSs-Pt	182	0.27 s^–1^	0.9 A mg_Pt_^–1^	11.2 μg cm^–2^
Pt	27			

Two well-distinct behaviors characterized the bare and Pt-decorated samples.

(i) Bare NSs presented very high overpotential, on the order of 400 mV vs RHE. Such high energies were required to overcome the H^+^ adsorption and successive H_2_ step production. Even if ZnO-based nanostructures are not really active for HER, we could note that our NSs presented promising results for HER in alkaline media, showing overpotential values significantly reduced in comparison to literature data (100 mV lower than in refs).^30,39^ Such an advantage could be attributed to defects present on the NS surface,^40,41^ as evidenced by photoluminescence analyses in our previous work.^33^

(ii) The presence of Pt NPs decorating NSs drastically reduced the overpotential from 485 to 180 mV, bringing up an enhanced catalytic action of Pt toward HER. For comparison, a Pt electrode was reported with an overpotential of 27 mV.

As far as the Pt decorated NSs are concerned, a huge reduction of the overpotential was gained. Still, the effect of Pt decoration must be properly quantified after the Pt loading mass. However, given the high surface exposure, NSs represented an efficient support for electrocatalysts.

The electrochemical surface area evaluation was carried out, and it is reported in Figure S3. Also, the LSV curves for both NSs and NSs-Pt-normalized ECSA are reported in Figure S4.

**Figure 4:**
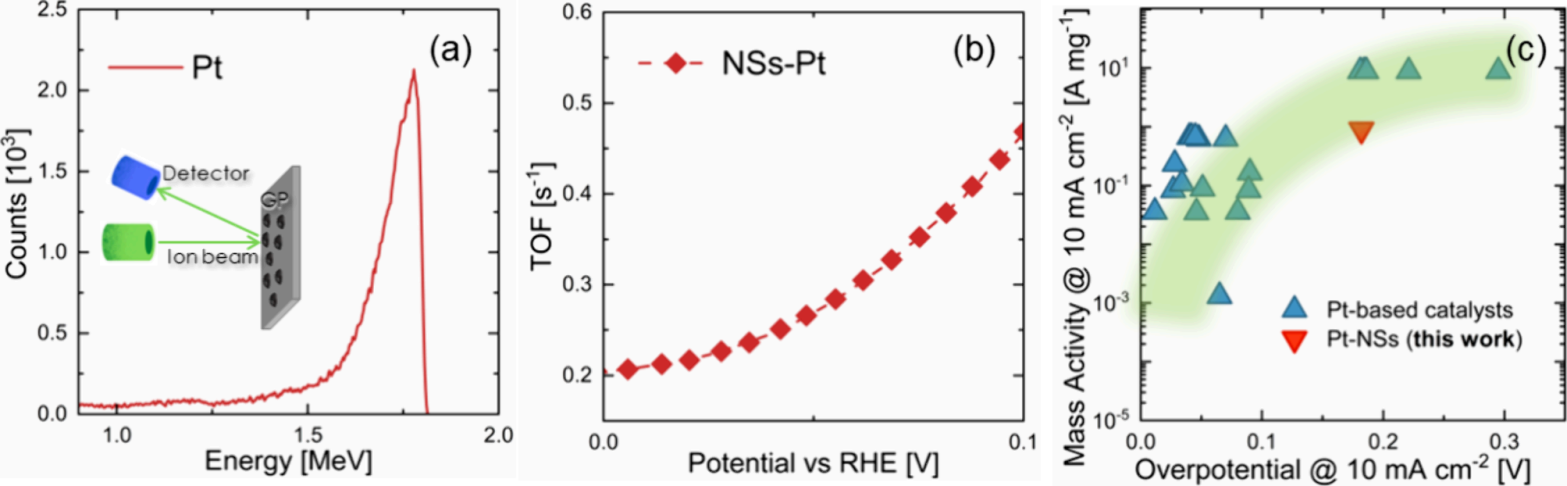
(a) RBS spectrum of Pt decoration performed on Si; (b) NSs-Pt TOF plots, in the range of 0–100 mV of overpotential, and (c) mass activity, with the comparison with state-of-the-art Pt-based electrodes.

Tafel plot curves are reported in Figure S5. In order to properly evaluate the amount of Pt loading on the NSs sample, RBS measurements were conducted on flat silicon substrates (Si) covered with the same amounts of drops of the electrodes. We assumed that after drop casting, the Pt mass on Si was the same as that on NSs. The RBS spectrum in Figure 4a proved the effective presence of Pt and enabled us to quantify the amount of platinum present on the substrate, as the Pt loading was associated to the area of the Pt peak in the spectrum.^42^

A Pt dose of 3.4 × 10^16^ cm^–2^ was evaluated, which means 11.2 μg cm^–2^. A remarkably low amount of Pt loaded onto the electrodes was evidenced with very promising HER performances, pointing out the great efficacy of our NSs as support for electrocatalysts.

Turnover frequency (TOF) represents a key parameter in the HER, as it provides the electrode intrinsic catalytic activity. Figure 4b shows the TOF, extrapolated from eq 7, in the range of 0–100 mV of overpotential. Pt-loaded NSs presented very high TOF values, comparable with many other Pt-based catalyzers.^40^

Figure 4c presents the mass activity (from eq 9), measured at 10 mA cm^–2^, as a function of the overpotential of our decorated electrode compared with other Pt-based catalysts under alkaline conditions present in the literature.^7^ Our sample showed quite high mass activity at moderately low overpotential, in good agreement with other Pt-based catalysts. It should be noted that further optimization of NSs could reduce the overpotential, as a significant potential drop could occur along the NSs.

**Figure 5:**
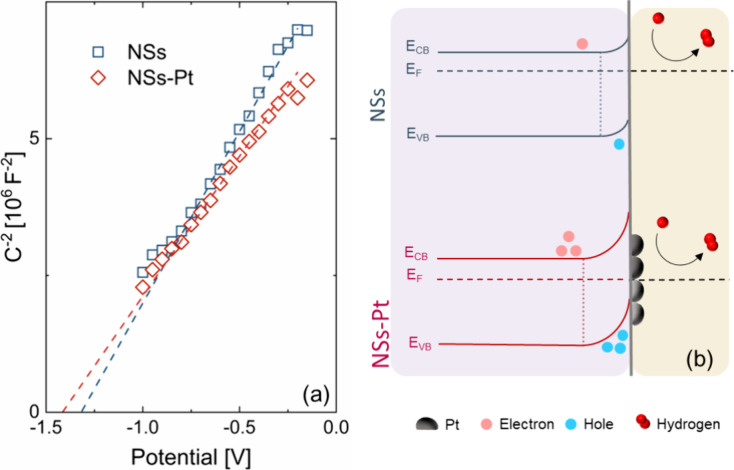
(a) NSs and NSs-Pt Mott–Schottky plots and (b) their schematic of energy levels at the surface.

To evaluate the effect of Pt decoration on ZnO NS, Mott–Schottky (M–S) analyses were performed. The Mott–Schottky plot for NSs and NSs-Pt are shown in Figure 5a. A typical M-S plot reports the inverse of the squared capacitance as a function of the potential applied to the sample. For an *n*-type semiconductor, as the applied potential decreases, the C^–^^2^ goes to zero.^43,44^ The intercept with the *x*-axis represents the flat band potential *V*_FB_ that needs to be corrected by the open circuit potential (*V*_OC_): Δ*V*_M–S_ = *V*_FB_ – *V*_OC_.^42^ Zinc oxide is an n-type semiconductor: once it is immersed in an electrolytic solution, the net effect arising from the alignment of the Fermi energy of the material and the redox potential of the electrolyte is the balancing of carriers at the solid–liquid interface. In particular, the semiconductor is depleted of electrons, leading to an accumulation of holes beneath its surface and to an upward semiconductor energy band bending. By modulating the applied voltage, it is possible to vary the degree of bending of the energy levels.^41^ The flat-band potential condition *V*_FB_ is reached when the applied potential is enough to flat the semiconductor bands independent of the position of the solution redox potential. Pt NP decoration clearly affected the position of the semiconductor energy levels at the ZnO-Pt interface, as proved by the Mott–Schottky analyses. Figure 5b summarizes the band bending in the case of bare NSs and NSs-Pt. Pt nanoparticles attracted the ZnO electrons at the interphase, an effect called “spillover”, preventing the electrode–electrolyte electron flow.^45,46^ By increasing the Pt amount, there was a slight increase in the band bending. Generally, a more negative CB position accounted for stronger reduction power, so the electrochemical performances improved with the Pt loading increase. Figure S6a–c shows the COMSOL simulations of the Pt-ZnO system (see the Supporting Information).

A series of electrochemical tests were also performed to evaluate the bare NS performances as a catalyst for the OER. All measurements were conducted in alkaline media (1 M KOH) as the HER tests. Figure S7 reports the polarization curve for NSs, obtained from the LSV. The LSV curve is normalized by the voltage drop, as for the HER curves, and the corresponding EIS spectra are reported in Figure S8. NSs possess an overpotential of 355 mV at a current density of 10 mA cm^–2^. This time, without any decoration, NSs presented a remarkable activity toward the OER, which is a significant achievement for a ZnO-based nanostructure.

The corresponding Tafel plot is reported in Figure S9.

As for the HER, the ECSA-normalized NS polarization curve is reported in Figure S10.

**Figure 6:**
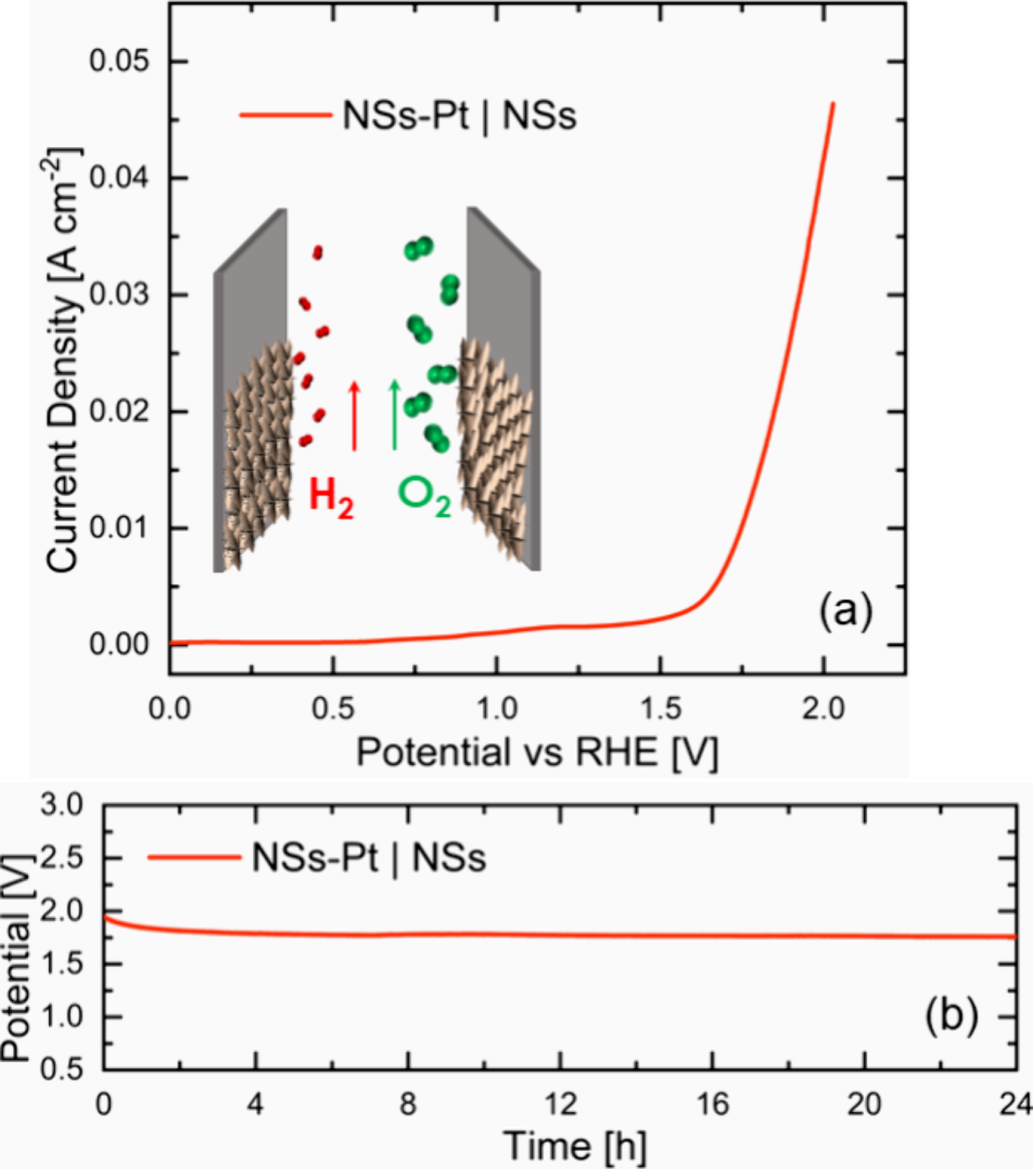
Overall water splitting (a) and stability (b) tests of the NSs-Pt || NSs cell.

Motivated by these data, an overall water splitting measurement in alkaline media was performed by using the NSs-Pt as a cathode and the bare NSs as an anode; a scheme of the cell used is in the inset of Figure 6a. The cell required a potential of 1.72 V to reach a current density of 10 mA cm^–2^ and 1.98 V to reach 40 mA cm^–2^, almost comparable to the commercial Pt/C || RuO_2_ standard cell, which has a potential of 1.63 V at 10 mA cm^–2^.

In addition, the cell stability was tested by performing a 24 h-long chronopotentiometry (Figure 6b), revealing very high stability over a prolonged time, even improving its performance with time. These last results confirmed that ZnO-Pt || ZnO electrodes represent very efficient electrocatalysts.

Figure S12 shows a Pt-decorated nanostar SEM image after the stability tests, in which it is evident how nanostars and Pt NP structures are maintained.

## Conclusions

We developed a highly effective electrocatalyst support for water splitting by synthesizing low-cost and high-yield ZnOH–ZnO nanostars. A decoration with an ultralow amount of Pt (11.2 μg cm^–2^) allowed us to measure an HER overpotential of 182 mV at 10 mA cm^–2^. The NS shape allowed us to maximize Pt utilization, as confirmed by the high values of mass activity (0.9 mA mg_Pt_^–1^) and turnover frequency (0.27 s^–1^) for a Pt loading of 11.2 μg cm^–2^. As far as the OER is concerned, bare NSs showed a remarkable value of 355 mV at 10 mA cm^–2^ in alkaline media, well lower than other ZnO-based nanostructures. Finally, our NSs showed promising performances as an alkaline, all ZnO-based electrolyzer, showing an overpotential of 1.7 V at 10 mA cm^–2^ and 1.98 for 40 mA cm^–2^, by using bare and Pt-decorated NSs (NSs-Pt || NSs) as the cathode and anode, respectively. Also, this cell was demonstrated to be very stable in time, with the potential at a current density of 10 mA cm^–2^ slightly decreasing with time. NSs demonstrated to be efficient electrocatalyst supports for HER, allowing to reach very high Pt mass activity. These cells based on abundant materials pave the way toward a new era of electrocatalysts for water splitting applications.

## Supporting Information Available

Additional experimental details, materials, and methods and images; NS low-magnification SEM image; Nyquist plot of NSs, Pt-NSs, and Pt; Δ*I* plot; HER LSV normalized by ECSA of NSs and Pt-NSs; Tafel plots of NSs, NSs-PT, and Pt; COMSOL simulations; polarization curve of NSs for OER; NS Nyquist plot; NS Tafel plot; OER NS LSV normalized by ECSA; NS XRD patterns; and Pt-NS SEM image after the 24 h stability test (PDF)
