# Supplementary material for: Low-Cost, High-Yield Zinc Oxide-Based Nanostars for Alkaline Overall Water Splitting
Source: ACS Omega. 2023 Sep 29;8(40):37023–31. doi: 10.1021/acsomega.3c03958 (PMC10568701; doi:10.1021/acsomega.3c03958)
Supplement: Supplementary file 1 — ao3c03958_si_001.pdf [file ao3c03958_si_001.pdf]

Supporting information

Low cost, high yield zinc oxide based nanostars for alkaline overall water splitting

Gisella Maria Di Mari [1,2], Maria Chiara Spadaro [3,4], Francesco Salutari[4], Jordi Arbiol[4,5], Luca Bruno [1,2], Giacometta Mineo[1,2], Elena Bruno [1,2] Vincenzina Strano [2] and Salvo Mirabella [1,2,*]

1 Dipartimento di Fisica e Astronomia "Ettore Majorana", Università degli Studi di Catania, via S. Sofia 64, 95123 Catania, Italy;

2 CNR-IMM, Catania (University) Unit, via S. Sofia 64, 95123 Catania, Italy;

3 Dipartimento SIMAU, Università Politecnica delle Marche, Piazza Roma 22, 60121, Ancona, Italy;

4 Catalan Institute of Nanoscience and Nanotechnology (ICN2), CSIC and BIST, Campus UAB, 08193 Bellaterra (Barcelona), Catalonia, Spain;

5 ICREA, Pg. Lluís Companys 23, 08010, Barcelona, Catalonia, Spain

* Corresponding author:

salvo.mirabella@dfa.unict.it (S. Mirabella)

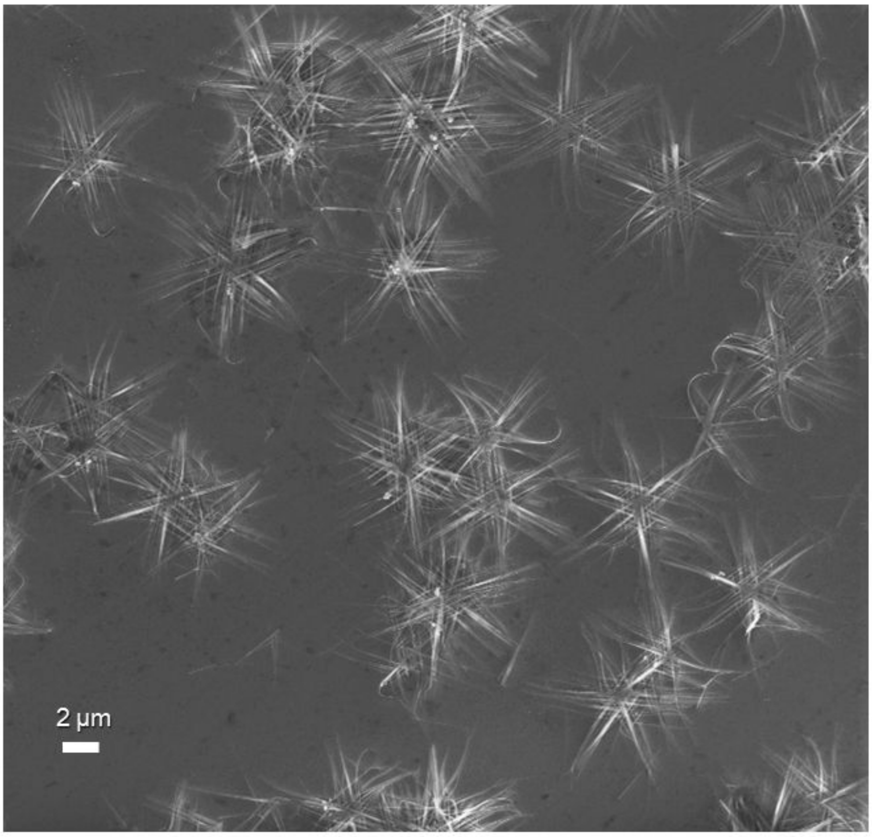

Figure S1 NSs low magnification SEM Image

The EIS spectra were obtained within the turnover region, immediately following the onset potential of each sample, to evaluate their HER activities [1]. The experimental EIS spectra were modeled using the Armstrong-Henderson equivalent circuit, represented by continuous lines [2].

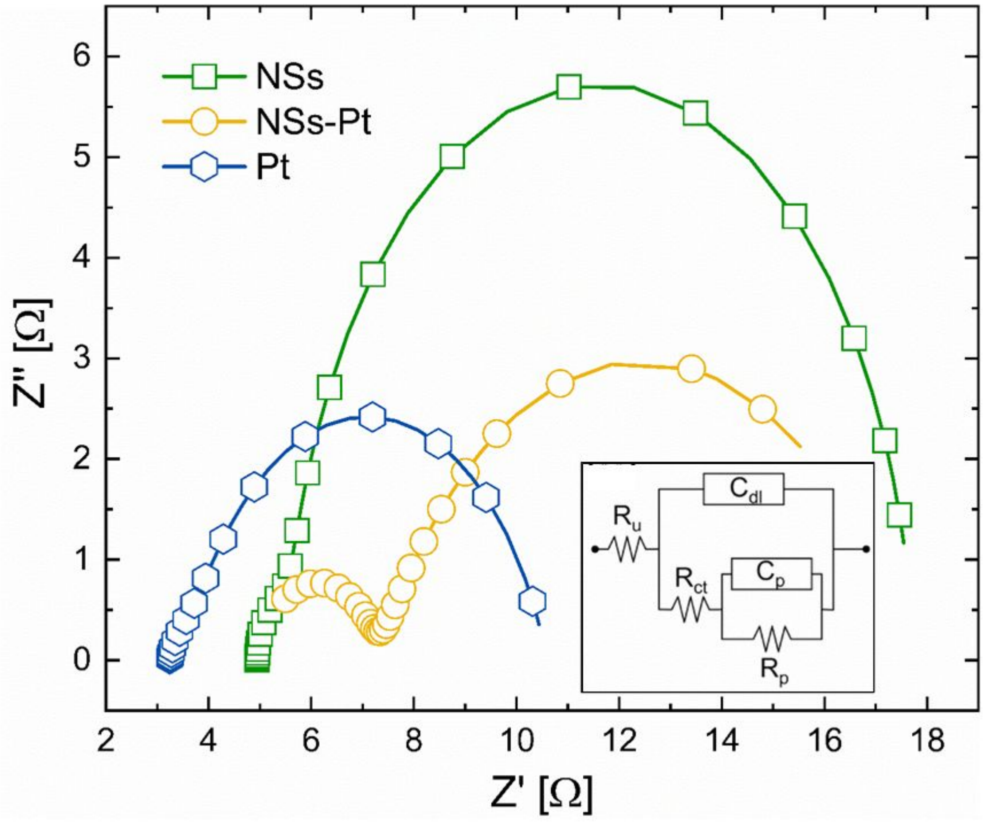

Figure S2 Nyquist plot of NSs, Pt-NSs and Pt (symbols). Full lines represent the fitted data, obtained fitting the curves by the Armstrong-Henderson equivalent circuit (inset).

The ECSA was measured by the double layer capacity (Cdl), following the equation:

ECSA = Cdl/Cs, where Cs is the specific capacitance for a flat surface; we assumed a value of Cs of 40 µF cm^{-2} [3].

For HER, Cdl was obtained from CV curves, at a potential ranging from -0.8 to 0.9 vs SCE, from the slope of (Ja-Jc) vs the scan rate plot at fixed potential (-0.85V) according to the follow relation [3]:

$$C_{dl} = (J_a - J_c)/2\nu$$

Our data presented an ECSA of 261.5 cm^2 for NSs-Pt and of 191 cm^2 for bare NSs. These very high ECSA values confirm the high surface exposed for bare NSs. Pt decoration slightly varied the ECSA value.

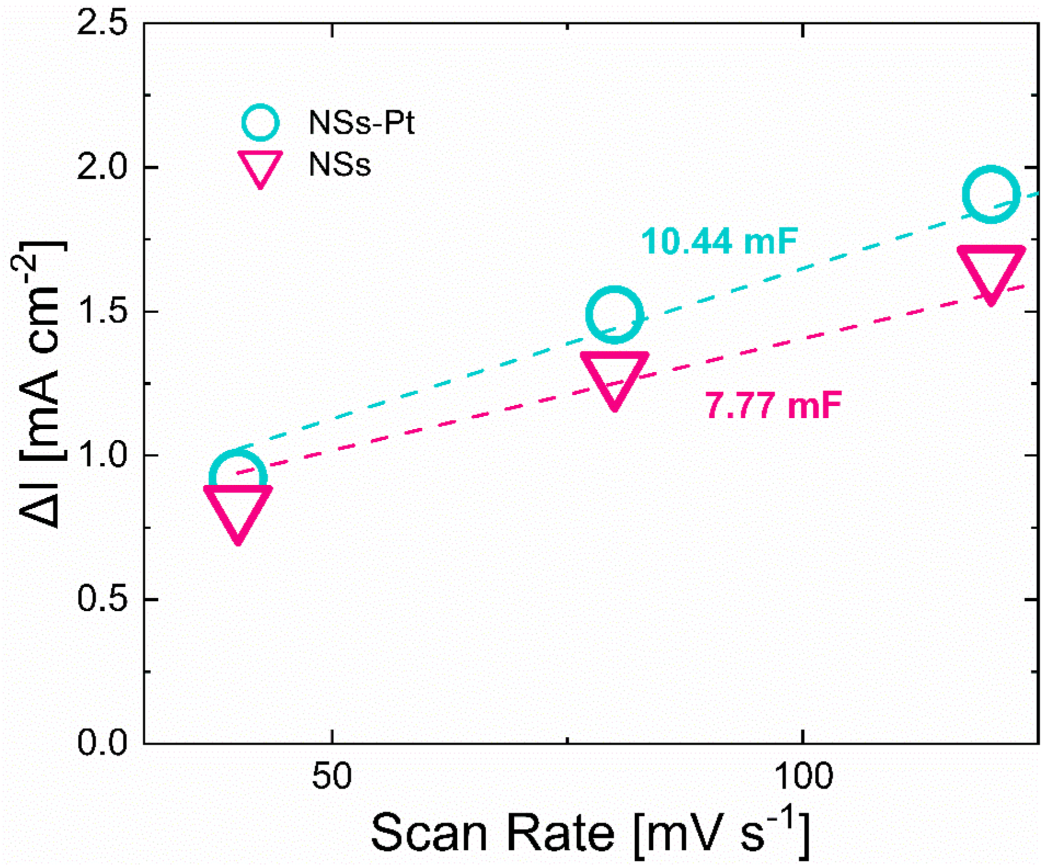

Figure S3 ΔI plot, recorded at a potential of -0.85V, vs scan rate (empty symbols), and the related fits (dashed lines)

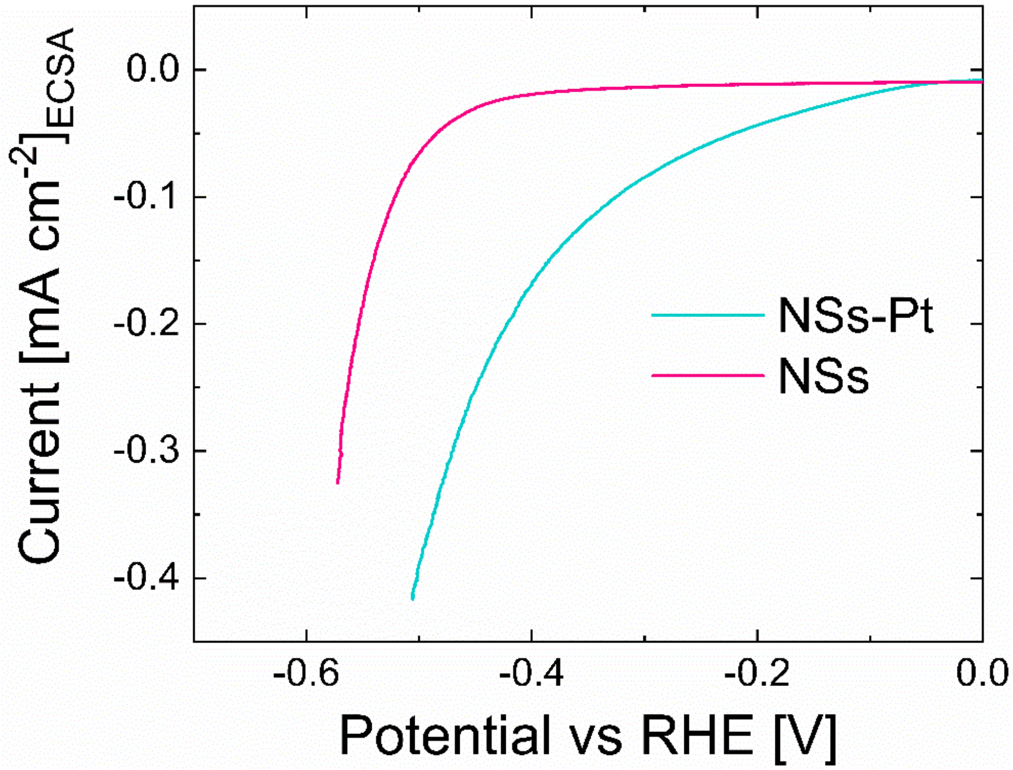

Figure S4 HER LSV normalized by ECSA of NSs and Pt-NSs

With Tafel slope it is possible to make considerations on the HER kinetic mechanism.

HER mechanism in high alkaline media involves three following steps [4]:

(i) water molecules electroreduction with hydrogen adsorption (H2O + e− → Hads + OH−), which is named Volmer reaction;

(ii) H_2 formation through electrochemical hydrogen desorption step (Hads + H_2O + e− → H_2 + OH−), which is named Heyrovsky step;

(iii) H_2 formation through a recombination step between two adsorbed hydrogen atoms (Hads + Hads → H_2), which is named Tafel step.

An evaluation of the rate-determining step (RDS) from Tafel slopes can be done [5]. Considering the Butler-Volmer equation as the foundation of kinetic analysis and mechanism, our values suggest that the RDS for our samples is represented by the Volmer step, meaning the initial hydrogen adsorption and water molecule electroreduction step.

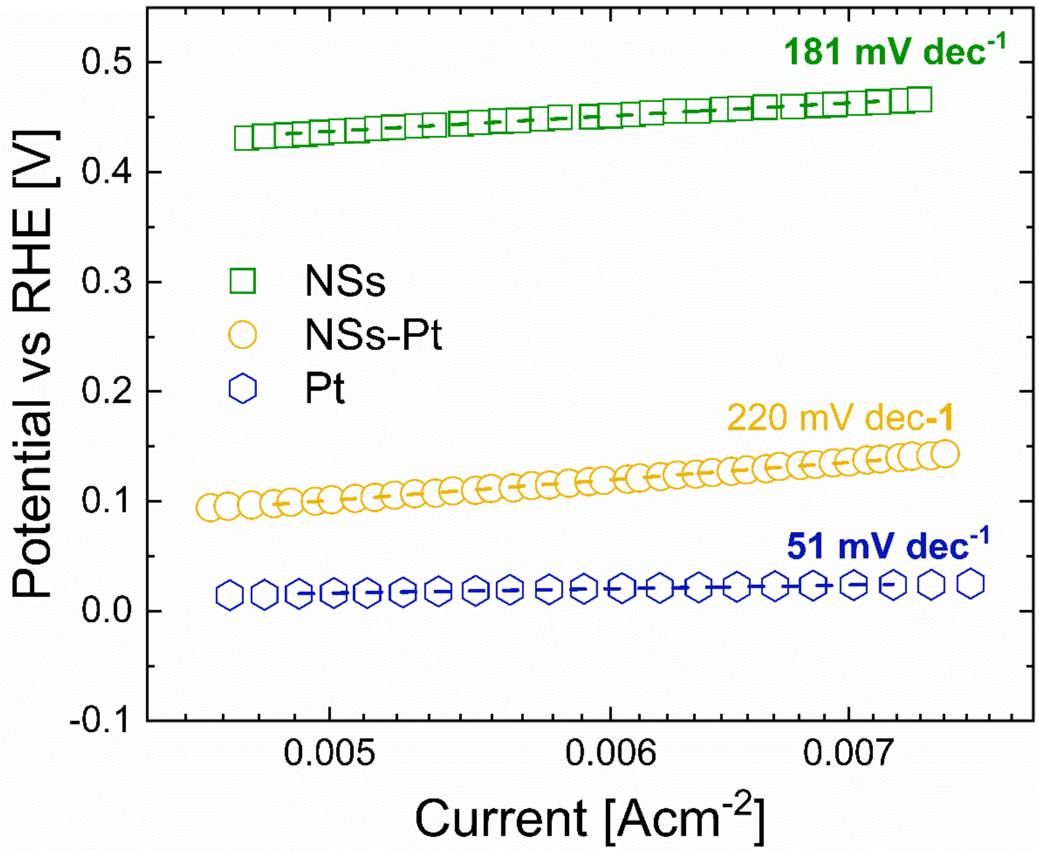

Figure S5 Tafel plots of NSs, NSs-PT and Pt.

We investigated the band bending at the metal-semiconductor interface via a multiphysics approach. COMSOL [6] simulations were performed, assuming a single Pt circular dot (50 nm as diameter) placed onto ZnO in a vacuum ambient. Band bending occurs at the Pt-ZnO interface because of the different work functions. Indeed, Pt has a higher work function (Φ_{Pt} = 6.35 eV) than ZnO (Φ_B = 4.2 eV) [7, 8] and such difference leads to a potential barrier for electrons and to a significant upward bending of the valence and conduction bands at the Pt-ZnO interface. The electric field 2D map at the NSs surface below a circular Pt dot is reported in Figure S2(a), showing intensity as high as $5 \times 10^7\ V\,m^{-1}$. This localized electric field caused by Pt decoration is extremely efficient in modifying the band levels and the carrier density at the surface. Finally, from the electric field we extrapolated the hole and electron concentrations, reported in Figure S2(b-c).

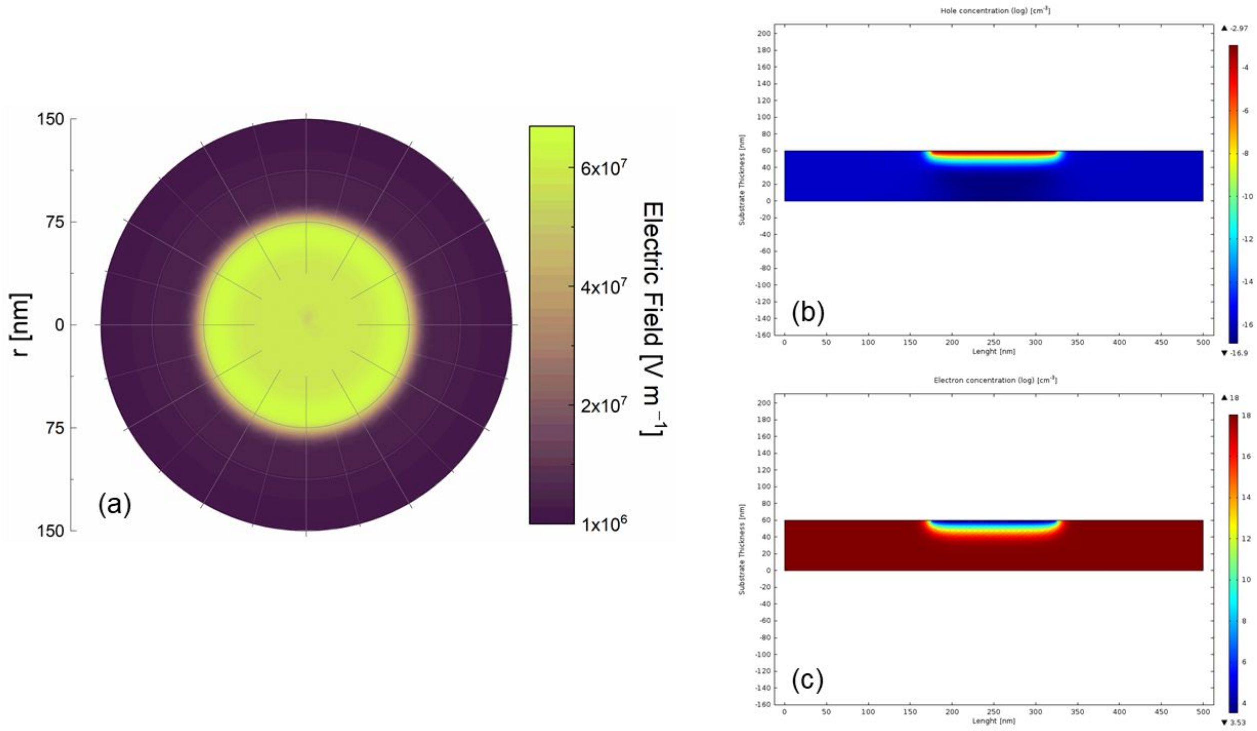

Figure S6 COMSOL simulations; (a) Electric field 2D map at the NSs surface below a circular Pt dot; (b) hole and (c) electron concentration profiles.

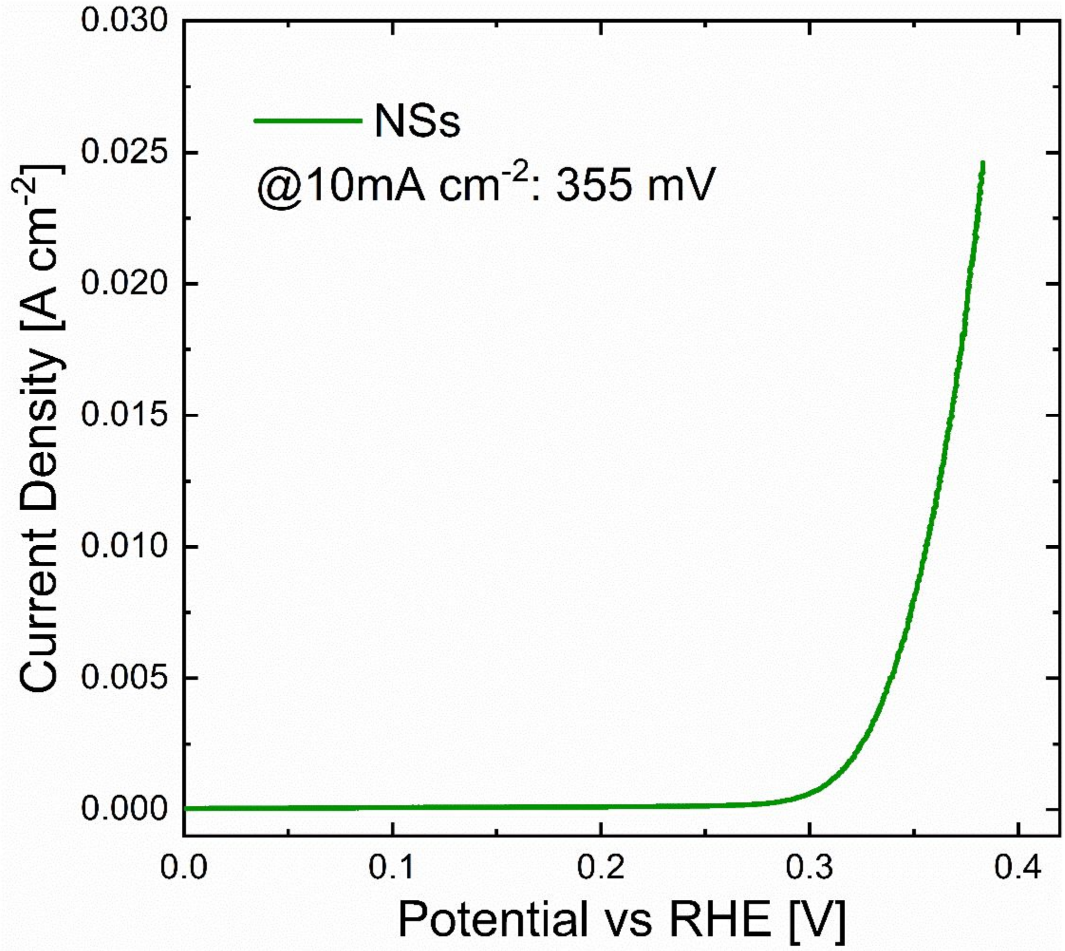

Figure S7 Polarization curve of NSs for Oxygen Evolution Reaction.

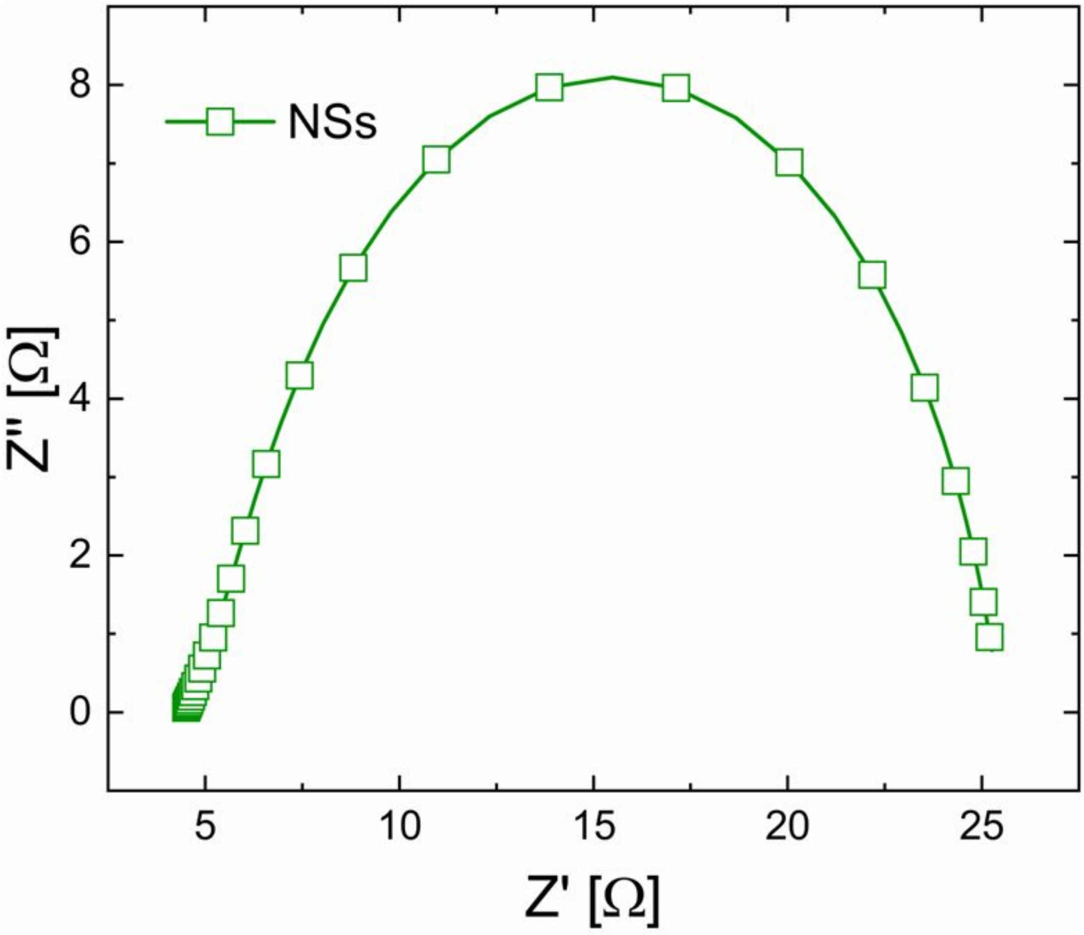

Figure S8 NSs Nyquist plot (symbols). The full line represents the fitted data, obtained fitting the curve by the Armstrong-Henderson circuit (see Figure S1)

The catalytic activity of NSs for OER wad also evaluated by Tafel Plot, extrapolated from the LSV. As for the HER, from the Tafel slope it is possible to make some considerations on the rate determining step. Our electrode presents a Tafel Slope of 49 mV dec^{-1}, which is usually related to an RDS consisting of the adsorption of OH^- ions on the surface of the electrocatalyst [4].

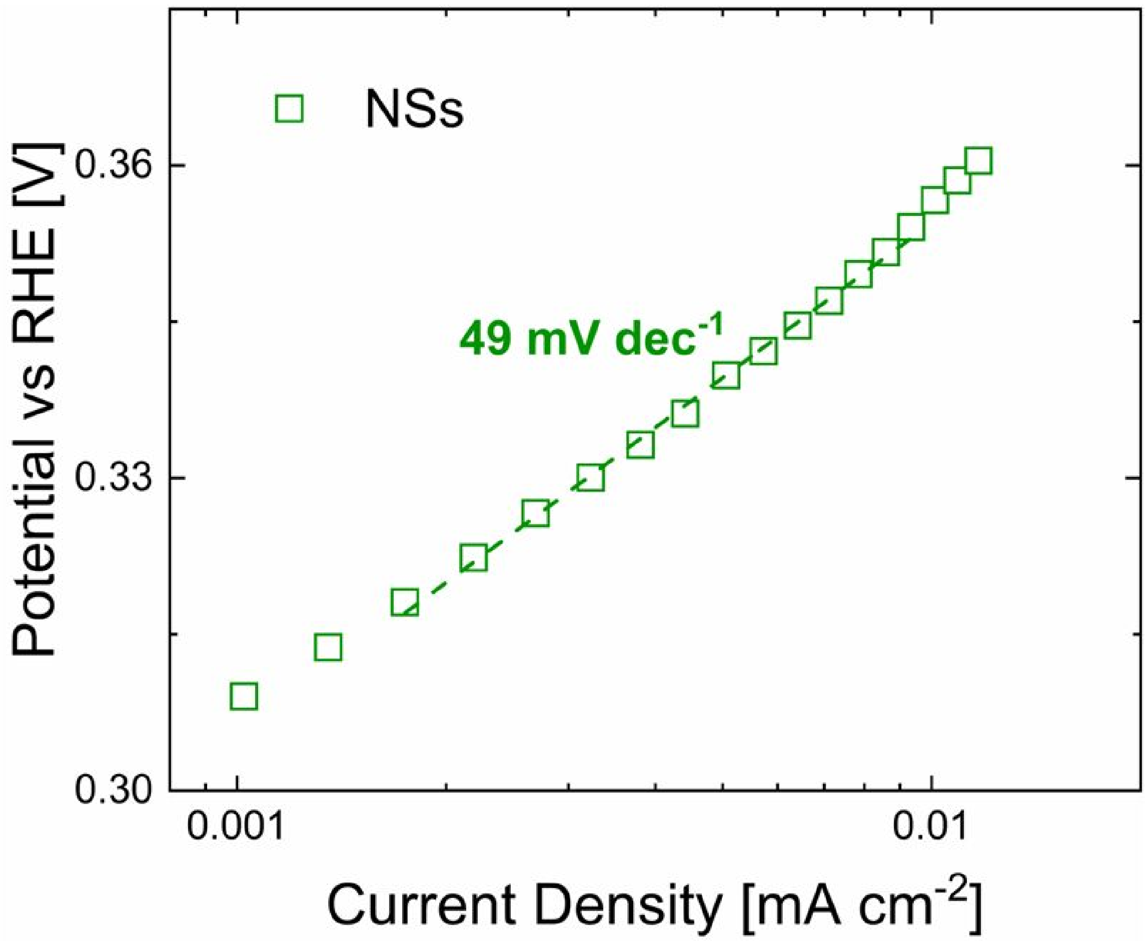

Figure S9 NSs Tafel plot.

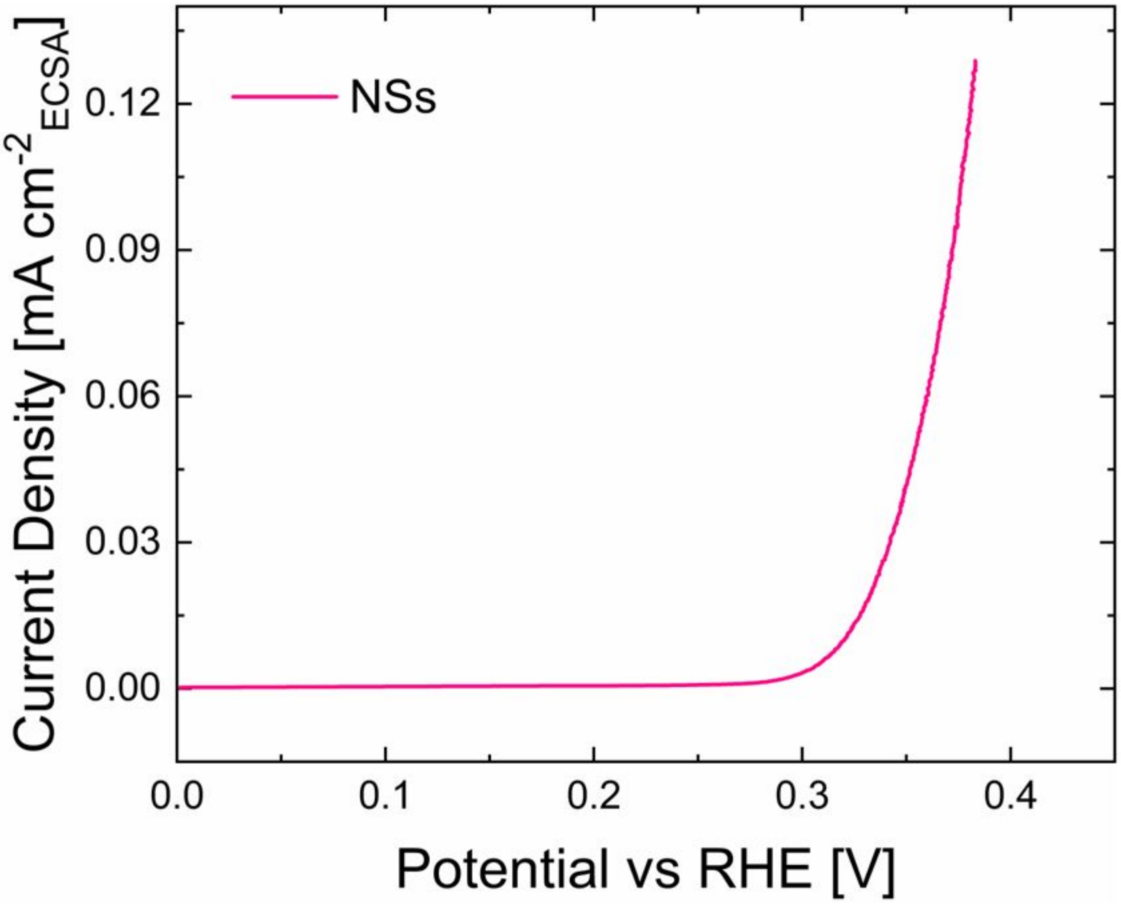

Figure S10 OER NSs LSV normalized by ECSA.

For OER, Cdl was obtained from CV curves, at a potential ranging from 0 to 0.1 vs SCE, from the slope of (Ja-Jc) vs the scan rate plot at fixed potential (0.05 V) according to the previous relation reported for HER. Our data presented an ECSA of 52 cm^2 for bare NSs.

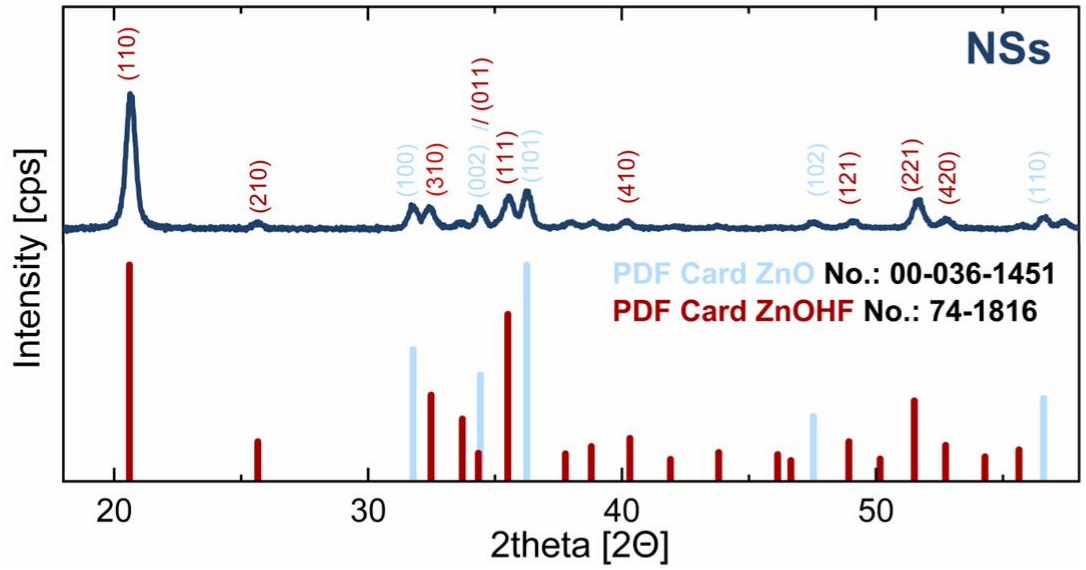

Figure S11 NSs XRD pattern

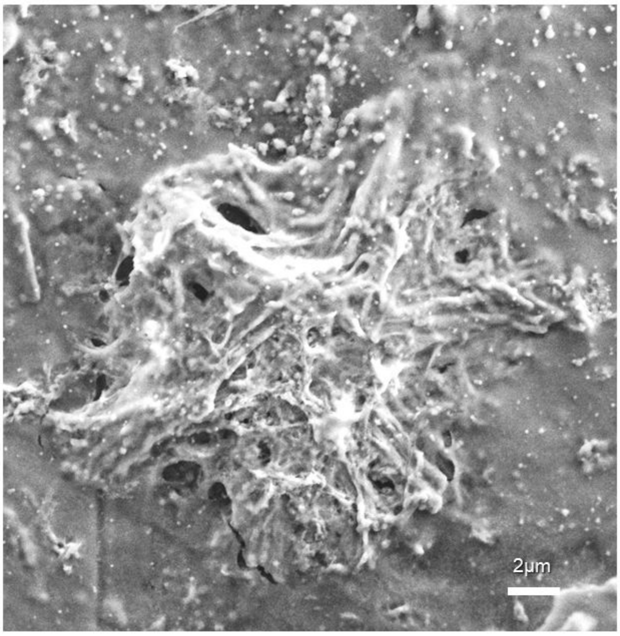

Figure S12 Pt-NSs SEM image after the 24h stability test

References

[1] S. Anantharaj, S. Noda, Appropriate Use of Electrochemical Impedance Spectroscopy in Water Splitting Electrocatalysis. ChemElectroChem (2020), 7 (10), 2297–2308. https://doi.org/10.1002/celc.202000515.

[2] R. D.Armstrong, M. Henderson, 176De904-8429-41E9-80E9-B81E414Eb64B. 1972, 39 (5).

[3] G. Mineo, M. Scuderi, E. Bruno, S. Mirabella, Engineering Hexagonal/Monoclinic WO3Phase Junctions for Improved Electrochemical Hydrogen Evolution Reaction. ACS Appl. Energy Mater. (2022), 5 (8), 9702–9710. https://doi.org/10.1021/acsaem.2c01383.

[4] F. Safizadeh, E. Ghali, G. Houlachi, Electrocatalysis Developments for Hydrogen Evolution Reaction in Alkaline Solutions - A Review. Int. J. Hydrogen Energy (2015), 40 (1), 256–274. https://doi.org/10.1016/j.ijhydene.2014.10.109.

[5] T. Shinagawa, A. T. Garcia-Esparza, K. Takanabe, Insight on Tafel Slopes from a Microkinetic Analysis of Aqueous Electrocatalysis for Energy Conversion. Sci. Rep. (201) 5, 1–21. https://doi.org/10.1038/srep13801.

[6] Index @ Www.Comsol.Com, (n.d.). https://www.comsol.com/.

[7] M. Alnot, J.J. Ehrhardt, J.A. Barnard, A characterization of heterogeneous Pt surfaces by work function measurements and photoemission of adsorbed xenon, Surf. Sci. 208 (1989) 285–305. https://doi.org/10.1016/0039-6028(89)90004-6.

[8] M.J. Al-Saadi, S.H. Al-Harthi, H.H. Kyaw, M.T.Z. Myint, T. Bora, K. Laxman, A. Al-Hinai, J. Dutta, Influence of Atomic Hydrogen, Band Bending, and Defects in the Top Few Nanometers of Hydrothermally Prepared Zinc Oxide Nanorods, Nanoscale Res. Lett. 12 (2017). https://doi.org/10.1186/s11671-016-1800-3.
